# Predictive value of hormonal parameters for live birth in women with unexplained infertility and male infertility

**DOI:** 10.1186/1477-7827-11-61

**Published:** 2013-07-11

**Authors:** Tiina Murto, Kerstin Bjuresten, Britt-Marie Landgren, Anneli Stavreus-Evers

**Affiliations:** 1 Department of Women’s and Children’s Health, Uppsala University, Uppsala, Sweden; 2 Department of Clinical Sciences, Intervention and Technology (CLINTEC), Karolinska Institute, Stockholm, Sweden

**Keywords:** AMH, FSH, Inhibin B, LH, Oestrogen, Progesterone, Prolactin, TSH

## Abstract

**Background:**

Infertile women might get pregnant sometime after fertility treatment, but today, there is no prediction model on who will eventually have children. The objective of the present study was to characterize hormone levels in an arbitrary menstrual cycle in women with unexplained infertility and male infertility, and to determine the predictive value for long-term possibility of live birth.

**Methods:**

In this cross-sectional study, with 71 infertile women with diagnosis unexplained infertility and male infertility, blood samples were obtained during the proliferative and secretory phases of an arbitrary menstrual cycle. Serum concentrations of FSH, LH, AMH, inhibin B, estradiol, progesterone, PRL and TSH were determined. The predictive value of ovulation and hormonal analysis was determined by identifying the proportion of women with at least one live birth. Mann Whitney U test, chi2 test and Spearman’s correlation were used for statistical analysis. A value of p < 0.05 was considered statistically significant.

**Results:**

There were no differences in hormone values and live birth rates between women with unexplained infertility and male infertility. The best sole predictors of live birth were age of the women, followed by ovulatory cycle, defined as serum progesterone concentration of greater than or equal to 32 nmol/L, and a serum TSH concentration of less than or equal to 2.5 mIU/L. Combining the age with the ovulatory cycle and serum TSH less than or equal to 2.5 mIU/L or serum AMH greater than or equal to 10 pmol/L the predictive value was close to 90%.

**Conclusions:**

Age in combination with the presence of an ovulatory cycle and serum TSH or serum AMH is predictive for long-term live birth. The advantage of serum AMH compared with serum TSH is the very little variation throughout the menstrual cycle, which makes it a useful tool in infertility diagnosis.

## Background

The menstrual cycle reflects a complex combination of synchronized endocrine events in which the hypothalamus, the anterior pituitary and the ovaries are involved [1]. These events are necessary for successful oocyte development, ovulation, fertilization and implantation.

Gonadotrophin-Releasing Hormone (GnRH), Follicle-Stimulating Hormone (FSH) and Luteinizing Hormone (LH) control ovarian function through a sensitive feedback system. A high level of serum FSH in the early follicular phase, a consequence of reduced ovarian function, is predictive of impaired pregnancy outcome after infertility treatment [2] and it has been shown that a combination of low FSH levels and high LH levels can predict pregnancy outcome after infertility treatment [3].

Anti-Müllerian hormone (AMH) is a dimeric glycoprotein produced by granulosa cells from pre-antral and antral follicles. The main role of AMH is to inhibit follicular development from primordial to primary follicular stages. During the menstrual cycle, serum AMH levels are relatively stable [4]. AMH seems also to be a better predictor of pregnancy outcome after IVF treatment than other hormonal parameters [5,6].

Inhibin B, produced by granulosa cells in antral follicles, has been suggested as a marker of follicular growth. Low serum inhibin B levels have been related to elevated levels of FSH, which have been shown to have an association with decreased oocyte quality and fertility [4]. The levels of inhibin B vary more than AMH throughout the menstrual cycle. However, the combination of concentrations of AMH and inhibin B is predictive for pregnancy after infertility treatments [7].

Oestrogen plays important roles in oocyte maturation, embryo quality and fertilization [8,9]. The most important oestrogen is estradiol (E) [8], the serum level of which in combination with that of FSH, and age is predictive of pregnancy outcome after IVF treatment [10].

Progesterone (P) is the most important hormone for endometrial development, implantation and maintenance of pregnancy [11]. Progesterone support during the luteal phase has been shown to result in significantly better pregnancy rates as well as live birth rates after infertility treatment [12,13].

Thyroid hormones influence ovarian function directly and indirectly through elevated prolactin (PRL) and altered GnRH secretion. In women with thyroid dysfunctions, ovarian insufficiencies are common [14]. Elevated levels of serum PRL have been associated with menstrual disorders as a result of its restraining effect on pulsatile GnRH secretion as well as inhibition of FSH and LH release [15]. Furthermore, hyperprolactinaemia has been shown to be associated with ovulatory dysfunction [16] and both hypothyroidism and hyperprolactinaemia have been implicated in infertility. However, the association between pregnancy outcome and serum TSH levels is controversial. It has been shown that infertile women with elevated levels of serum TSH have lower pregnancy rates than women with normal TSH levels [16].

Approximately 10% of all couples worldwide are infertile [17]. In the western world, women tend to have their first child at an older age, which decreases overall fertility. Therefore, we believe that women with tendency of early ovarian failure will be the most vulnerable group of women in this respect. The most successful treatment of infertility is in vitro fertilization (IVF) [18] with pregnancy rates between 13–43% per single IVF cycle [18-20]. However, some couples experience repeated failures of pregnancy after treatment [21].

Women with unexplained infertility are often considered to be subfertile, and will eventually become pregnant spontaneously sometime after fertility treatment [22]. The possibility of successful pregnancy in the long term depends on the presence of ovulatory menstrual cycles. The long-term probability of live birth in women with unexplained infertility and women with male infertility has to our knowledge not previously been investigated. Therefore, we studied serum hormone levels in an arbitrary menstrual cycle and determined the predictive value for long-term possibility of live birth in women with unexplained infertility and male infertility.

## Methods

### Study design and subjects

Seventy-one infertile women, referred to the Fertility Unit at Karolinska University Hospital Huddinge between 1999 and 2008 participated in the study. Recruitment of the women was focused on unexplained infertility and male infertility, as these two groups of patients have normal menstrual cycles. All women were referred from an outpatient gynaecological clinic to the Fertility Unit for further examination and treatment of infertility. The women were healthy except for their infertility and did not use any hormonal medication.

Infertility was determined by way of a standard set of tests that included hormone analyses and at least two semen analyses. Tubal passage was demonstrated by Hysterosalpingo Contrast Sonography (HyCoSy), or, if needed, laparoscopy was performed to exclude factors such as endometriosis. Semen analyses were based on WHO criteria of normality. The final diagnosis was established after these examinations. The diagnosis of unexplained infertility was chosen when no explanation for infertility was found, and the diagnosis of male infertility was set for women whose partner had abnormal semen analyses.

Most of the women were contacted by telephone five years after inclusion in the study and asked about pregnancy outcome, or the data of live birth was determined from patient records. Informed consent was obtained from all participating women and the study was approved by the Ethics Committee of Karolinska Institutet.

### Collection of blood samples

Blood samples were obtained at regular intervals during one arbitrary normal menstrual cycle. In most women blood was collected three times during the follicular phase and four times during the luteal phase for assay of serum FSH, LH, AMH, inhibin B, E, P, PRL and TSH. All hormones except E and P were analysed in blood obtained at cycle day 2 to 5. Serum E levels were determined on three occasions; before the LH surge (LH -6 to LH -1), the early secretory phase (LH 0 to LH +5) and the mid-luteal phase (LH +6 to LH +9). Progesterone was assayed in four different samples during the luteal phase for determination of area under the curve (AUC), measured as concentration of progesterone/day. Additionally, the value determined during the mid-luteal phase (LH +6 to LH +8) is also shown.

### Hormone analyses

All blood samples were analysed at the same time using routine methods at the central clinical laboratory (FSH, LH, E, P and PRL) at Karolinska University Hospital Huddinge or at the research laboratory, Department of Obstetrics and Gynaecology, Uppsala University Hospital, Uppsala, Sweden (TSH, AMH and inhibin B).

Serum concentrations of FSH and LH were measured using AutoDELFIA hFSH and hLH respectively (Perkin Elmer, Waltham, Massachusetts, USA). An enzyme-linked immunosorbent assay (ELISA) (Immunotech, A Beckman Coulter Company, Marseille, France) was used to determine serum AMH levels. The intra-assay CV of the ELISA was 12.3% and the inter-assay CV was 14.2%. The active Inhibin B Gen II enzyme-linked immunosorbent assay (Diagnostic Systems Laboratories, Inc., A Beckman Coulter Company, Webster, USA) was used to measure serum inhibin B levels. The intra-assay CV of the ELISA was 2.40% and the inter-assay CV was 3.68%. Two values of inhibin B were determined to be < 7 pg/mL. These were set to 7.0 pg/mL in the statistical analysis. Serum E and P were assayed by using Modular Analytics E170 equipment (Roche, Basel, Switzerland). Levels of TSH and PRL were measured by using a Roche Cobas e601 system (Roche, Basel, Switzerland).

### Definition of ovulation, cut-off values and live birth

Ovulation was established according to Landgren et al. [23], using a serum P concentration of ≥ 32 nmol/L (anovulation < 32 nmol/L). For FSH < 7.0 IU/L, for the FSH:LH ratio ≤ 2.0 [24] and for AMH ≥ 10 pmol/L were used as cut-off values. The cut-off value for TSH was set to 2.5 mIU/L according to Baker et al. [25]. Serum concentrations of inhibin B and PRL varied considerably; therefore these hormones were not used for prediction of live birth. Live birth was defined as delivery of a healthy child. All live births were considered regardless of time in relation to the arbitrary cycle and regardless of whether or not it was spontaneously conceived or conceived after infertility treatment.

### Statistical analysis

Statistical analyses were performed using SPSS (Statistical Package for the Social Sciences) software (SPSS 15.0 for Windows; SPSS Inc. Chicago, IL). Data is given as median (range). We used the Mann Whitney U test for comparisons between two groups. For comparisons including categorical variables the χ^2^ test was applied. Spearman’s correlation was used for correlation analysis. A value of p < 0.05 was considered statistically significant.

The predictive value of ovulation and hormonal analysis was determined by identifying the proportion of women with at least one live birth 5 years or more after inclusion in the study. The calculation of the predictive value was performed as previously described [26].

## Results

### Patient characteristics

Patient characteristics are shown in Table 1. The groups consisted of 42 women with unexplained infertility and 29 women with male infertility.

**Table 1:** Characteristics of women included in the study

	**Unexplained infertility**	**Male infertility**	**P-value**
	**n=42**	**n=29**	
*Age years*	34 (26–41)	34 (29–39)	0.991
*BMI (kg/m*^*2*^*)*	22 (18–32)	22 (18–30)	0.670
*Age at menarche*	13 (10–17)	13 (12–17)	0.627
*Menstrual duration (Days)*	5 (4–7)	5 (3–8)	0.746
*Menstrual cycle length*	28 (24–35)	28 (25–36)	0.781

Data is shown as median and range.

### Hormone profile and pregnancy outcome in relation to diagnosis

There were no significant differences in hormone values or pregnancy outcome in relation to diagnosis when comparing the women with unexplained infertility and women with male infertility. Hormone concentrations and pregnancy outcome in relation to infertility diagnosis are shown in Table 2.

**Table 2:** Serum hormone levels, ovulation and total live births according to infertility diagnosis

	**Unexplained infertility**	**Male infertility**	**P-value**
*LH (IU/L)*	4.4 (1.5 - 11.0) n=42	3.8 (1.0 – 13.0) n=29	0.158
*FSH (IU/L)*	7.2 (3.4 – 23.0) n=42	6.1 (2.5 – 12.0) n=29	0.062
*FSH:LH ratio*	1.7 (0.5 – 4.2) n=42	1.6 (0.5 – 5.8) n=29	0.949
*AMH (pmol/L)*	19.3 (1.3 – 60.8) n=42	21.1 (5.3 – 60.8) n=29	0.977
*Inhibin B (pg/ml)*	37.1 (7.0-95.4) n=41	47.5 (13.0 – 138.4) n=27	0.208
*Estradiol LH-6 to LH-1 (pmol/L)*	596.0 (186–2330) n=26	675.5 (217–2924) n=22	0.203
*Estradiol LH+0 to LH+5 (pmol/L)*	661.5 (209–2511) n=40	579.0 (240–3117) n=25	0.535
*Estradiol LH+6 to LH+9 (pmol/L)*	434.5 (233–2063) n=40	450.5 (235–2053) n=28	0.727
*Progesterone, LH+6 to LH+8 (nmol/L)*	35 (20 – 66) n=37	42.5 (15–71) n=26	0.121
*Progesterone AUC nmol/L/day*	204 (33–504) n=37	229 (46 – 410) n=24	0.241
*Ovulatory cycle: P ≥32 (nmol/L)*	28 of 42 (66.7%)	24 of 29 (82.8%)	0.218
*PRL (μg/L)*	10.8 (3.8 – 28.0) n=41	11.8 (3.8 – 29.0) n=29	0.971
*TSH (mIU/L)*	1.3 (0.1 – 3.6) n=41	1.5 (0.4 – 7.7) n=29	0.535
*Total live births (%)*	20 of 41 (48.8%)	17 of 29 (58.6%)	0.569

Data is shown as median and range and n (%).

### Ovulation

Lack of ovulation could be a sign of premature menopause. The occurrence of ovulation did not affect any of the other hormones or the possibility of a live birth after 5 years or more. The numbers of women that ovulated in both groups are shown in Table 2.

### Correlations

**Figure 1:**
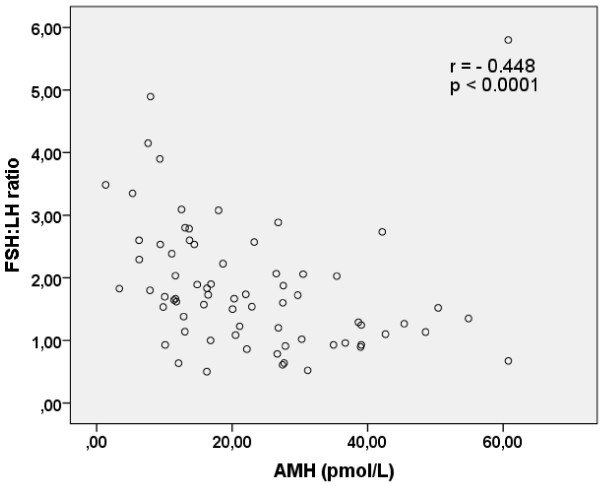
**Correlation between serum AMH and the FSH:LH ratio.** Correlation between serum AMH levels and the FSH:LH ratio in 71 infertile women with diagnosis unexplained infertility and male infertility. Spearman’s correlation analysis was used. A value of p < 0.05 was considered statistically significant.

Serum levels of AMH were associated with the FSH:LH ratio (p < 0.001) (Figure 1), but not with those of FSH, (p = 0.11). Serum levels of inhibin B were associated with those of AMH (p = 0.013), but not with those of FSH, or the FSH:LH ratio (p = 0.266 and 0.112 respectively).

### Prediction of pregnancy outcome

None of the studied hormones were good predictors of long-term pregnancy outcome (Table 3). The best hormonal predictor, with a predictive value of 56%, sensitivity of 78% and specificity of 30% was the presence of ovulatory menstrual cycles (Table 3). However, and not surprisingly, age was the best predictor of successful future pregnancies – predictive value 77%, sensitivity 54% and specificity 82% (Table 3). Combined prediction using the three best predictive values: age, presence of ovulatory cycles as determined by P ≥ 32 nmol/L and TSH ≤ 2.5 mIU/L increased the probability of predicting live birth to 88%, with a sensitivity of 100% and specificity of 33%. Since AMH has increased in popularity in infertility evaluation, we also calculated a combined predictive value of age, presence of ovulatory cycle and AMH ≥ 10 pmol/L. This calculation resulted in the predictive value of 83%, sensitivity of 83% and specificity of 25%. Menstrual cycle length, and serum inhibin B and PRL concentrations did not predict the later possibility of a live birth (data not shown).

**Table 3:** Live birth after infertility treatment in 71 infertile women in relation to different predictors

		**Ovulation **^**a)**^		**FSH **^**b)**^		**FSH:LH ratio **^**b)**^		**AMH **^**b)**^		**TSH **^**b)**^		**Age **^**b)**^	
*Live birth*		+	-	+	-	+	-	+	-	+	-	+	-
*Test result*	Normal	29	23	20	18	26	23	30	30	34	28	20	6
	Abnormal	8	10	17	15	11	10	7	3	2	5	17	27
*Sensitivity%*		78.4		54.1		70.3		81.1		94.4		54.1	
*Specificity%*		30.3		45.5		30.3		9.1		15.2		81.8	
*Predictive value%*		55.8		52.6		53.1		50.0		54.8		76.9	

^a)^ Ovulation was determined by using progesterone value as an indicator, ≥ 32 nmol/L being considered to represent ovulation (normal).

^b)^ Cut-off values (“Normal”) were determined to be FSH < 7 IU/L, FSH:LH ratio ≤ 2.0, AMH ≥ 10 pmol/L, TSH ≤ 2.5 mIU/L and age ≤ 32 years.

## Discussion

Serum hormone concentrations in 71 women, with diagnoses unexplained infertility and male infertility, were studied during one arbitrary natural menstrual cycle to investigate the possibility of predicting live birth. There were no significant differences in hormone levels or pregnancy outcome between women with unexplained infertility and women with male infertility.

One reason for increased infertility problems in Sweden is that women tend to delay their pregnancies, which results in a reduced fertility rate [27]. This is also seen in the present study where it is shown that women at the age of 32 or less have the highest chance of a future live birth. Age of the women had the best predictive value of live birth, which is also known from previous studies [28,29]. The increasing age has also been associated with a shortening length of the menstrual cycle, and the women with cycle length < 26 days has been shown to have significantly lower pregnancy rates after infertility treatment compared with the women with cycle length >34 days [30]. However, this could not been shown in our study, where the menstrual cycle length was not related to the pregnancy outcome.

The presence of one progesterone value ≥ 32 nmol/L was indicative of ovulation [23]. In the present study, an ovulatory cycle as assessed by assay of serum P was a better predictor of a future live birth than levels of any of the other hormones. It has previously been suggested that women with unexplained infertility have diminished ovarian reserve [31], but this was not noticed in our study.

The use of AMH for determination of ovarian function instead of FSH and/or FSH:LH ratio has increased, and therefore we studied the correlations between these parameters. Serum concentrations of AMH correlated with the FSH:LH ratio, and also with inhibin B levels, which leads to the conclusion that AMH could replace these markers in assessment of ovulatory function. The advantage of AMH is its capacity to maintain relative stable levels during the menstrual cycle [32] although circadian variations have been observed [33]. The disadvantage of AMH is that there are still no international standard assays for AMH measurement, which makes comparison between different laboratories complicated [34].

It has previously been shown that FSH:LH ratio can be used as predictor of pregnancy outcome in infertile women [35], but this was not confirmed in the present study. It has also been demonstrated that AMH is a better marker than age, FSH on cycle day 3 or inhibin B for prediction of IVF pregnancy success [36], which could neither be shown in the present study. However, our data showed that AMH combined with age and an ovulatory menstrual cycle was predictive for future live birth.

There are only limited data on inhibin B and AMH, and we are not aware of any studies in which groups of infertile women have been compared. However, there was considerable individual variation in serum levels of inhibin B and PRL, which shows that these hormones are not useful in diagnosis and prediction of live birth in women with unexplained and male infertility.

We found no differences in TSH levels between women in the studied groups. Previously, Cramer et al. [16] demonstrated relatively high TSH levels and relatively low PRL levels in women with male infertility. Conversely, Arojoki et al. [37] found the lowest TSH levels in women with male infertility, and the highest levels of TSH in women with unexplained infertility, and ovulatory dysfunction. However, menstrual disorders in women with hypothyroidism are more rare than previously reported [38]. In our study TSH as a single variable was not predictive for future live birth, but the combination with age of 32 or less, ovulatory cycle and TSH ≤ 2.5 mIU/L resulted in a predictive value of 88%.

The strength of the present study is the number of hormones measured. Eight different hormones were measured at various occasions during the menstrual cycle and the patients needed to come to the clinic several times during one natural cycle, which may have been challenging for women undergoing in vitro fertilisation. Additionally, our study group was well-defined in two selected groups; unexplained infertile and male infertility, while many previous studies have included patients regardless of cause of infertility. To our knowledge, the long-term probability of live birth in women with unexplained infertility and women with male infertility has not previously been studied.

A weakness of our study is the long recruitment period, which resulted in a prolonged follow-up time. Possible factors (e.g. stress) influencing the serum hormone levels were not taken into account, which may be considered a drawback.

## Conclusions

It can be concluded from the present results that there are no perfect markers for prediction of pregnancy outcome on a long-term basis; the best is still the age of the woman. Age in combination with the presence of an ovulatory cycle and a serum TSH concentration of ≤ 2.5 mIU/L or AMH concentration of ≥ 10 pmol/L is predictive of live birth in the long term. The advantage of serum AMH is its only slight variation in concentration throughout the menstrual cycle, which makes it a more useful tool in infertility diagnosis than TSH.

## Competing interests

The authors declare that they have no competing interests.

## Authors’ contributions

TM participated in the design of the study and its coordination, and performed the statistical analysis and drafted the manuscript. KB carried out the patient recruitment and collected the blood samples, and participated in the coordination of the study. BML conceived of the study and participated in its design and coordination, and helped to draft the manuscript. ASE participated in the design of the study and its coordination, and helped to perform the statistical analysis and drafted the manuscript. All authors read approved the final manuscript.
